# Variable δ^15^N Diet-Tissue Discrimination Factors among Sharks: Implications for Trophic Position, Diet and Food Web Models

**DOI:** 10.1371/journal.pone.0077567

**Published:** 2013-10-17

**Authors:** Jill A. Olin, Nigel E. Hussey, Alice Grgicak-Mannion, Mark W. Fritts, Sabine P. Wintner, Aaron T. Fisk

**Affiliations:** 1 Great Lakes Institute for Environmental Research, University of Windsor, Windsor, Ontario, Canada; 2 Warnell School of Forestry and Natural Resources, University of Georgia, Athens, Georgia, United States of America; 3 KwaZulu-Natal Sharks Board, Umhlanga Rocks, South Africa; 4 Biomedical Resource Unit, University of KwaZulu-Natal, Durban, South Africa; University of California Davis, United States of America

## Abstract

The application of stable isotopes to characterize the complexities of a species foraging behavior and trophic relationships is dependent on assumptions of δ^15^N diet-tissue discrimination factors (∆^15^N). As ∆^15^N values have been experimentally shown to vary amongst consumers, tissues and diet composition, resolving appropriate species-specific ∆^15^N values can be complex. Given the logistical and ethical challenges of controlled feeding experiments for determining ∆^15^N values for large and/or endangered species, our objective was to conduct an assessment of a range of reported ∆^15^N values that can hypothetically serve as surrogates for describing the predator-prey relationships of four shark species that feed on prey from different trophic levels (i.e., different mean δ^15^N dietary values). Overall, the most suitable species-specific ∆^15^N values decreased with increasing dietary-δ^15^N values based on stable isotope Bayesian ellipse overlap estimates of shark and the principal prey functional groups contributing to the diet determined from stomach content analyses. Thus, a single ∆^15^N value was not supported for this speciose group of marine predatory fishes. For example, the ∆^15^N value of 3.7‰ provided the highest percent overlap between prey and predator isotope ellipses for the bonnethead shark (mean diet δ^15^N = 9‰) whereas a ∆^15^N value < 2.3‰ provided the highest percent overlap between prey and predator isotope ellipses for the white shark (mean diet δ^15^N = 15‰). These data corroborate the previously reported inverse ∆^15^N-dietary δ^15^N relationship when both isotope ellipses of principal prey functional groups and the broader identified diet of each species were considered supporting the adoption of different ∆^15^N values that reflect the predators’ δ^15^N-dietary value. These findings are critical for refining the application of stable isotope modeling approaches as inferences regarding a species’ ecological role in their community will be influenced with consequences for conservation and management actions.

## Introduction

 Stable isotope analyses have proven to be a powerful tool for characterizing trophic relationships between predator and prey across ecosystems. An important parameter necessary to interpret stable isotope data in ecology is the diet-tissue discrimination factor (∆^15^N or ∆^13^C)—the enrichment in the heavy isotope in predators’ tissues relative to the prey consumed due to preferential assimilation of the heavy isotope and preferential excretion of the light isotope [1,2]. Following an influential paper by Post [3], the across-taxa mean enrichment of 3.4‰ remains the most broadly applied ∆^15^N value [4]. Controlled feeding experiments, however, have demonstrated that ∆^15^N values vary among species [5,6], with meta-analyses and experimental work empirically showing an inverse relationship between ∆^15^N and dietary δ^15^N values [7,8]. These data suggest that taxonomic classes containing a range of species that feed on diverse prey resources will exhibit intra-class variation in ∆^15^N, questioning the suitability of a single ∆^15^N value for characterizing predator-prey relationships [7,9]. 

**Figure 1:**
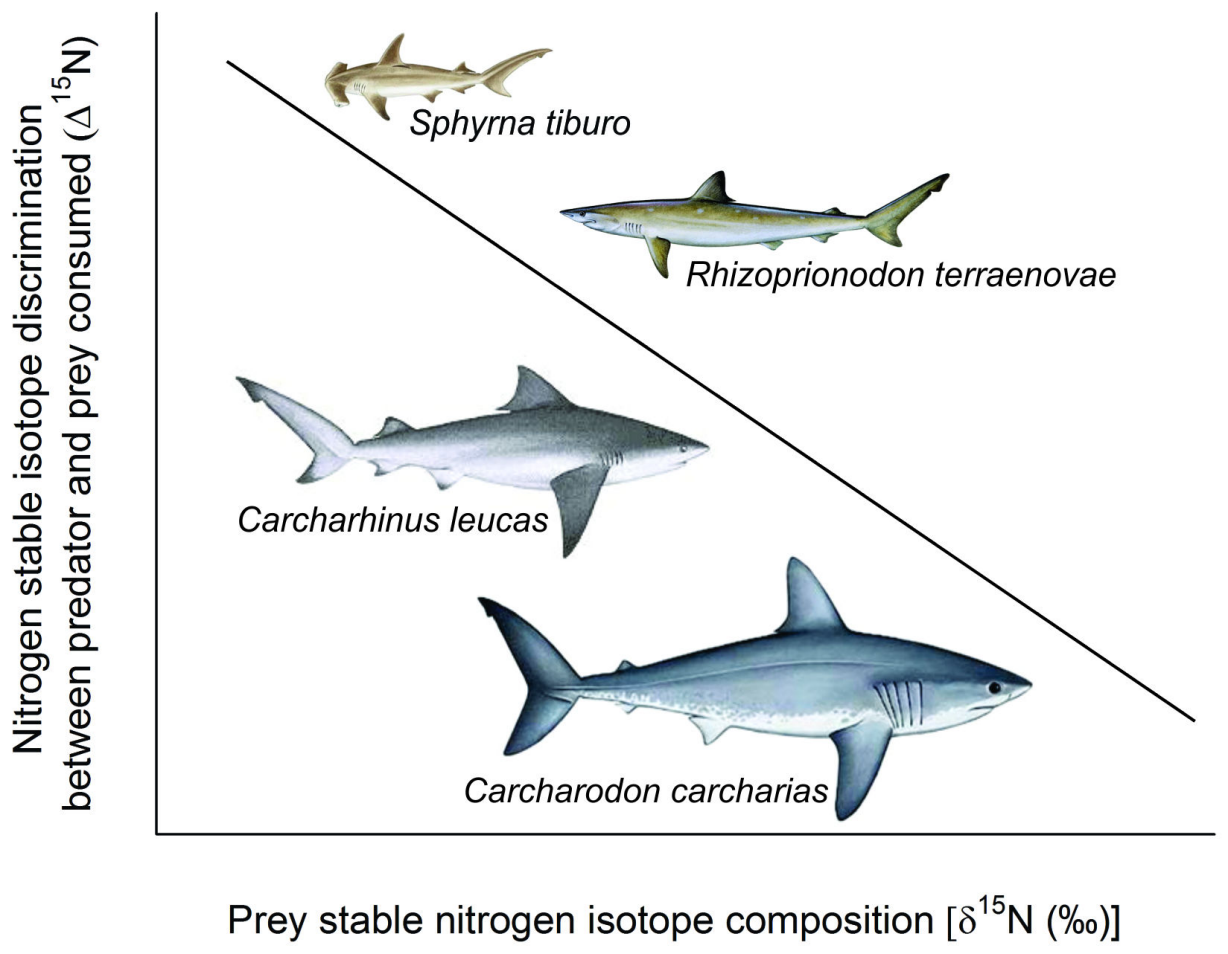
The predicted relationship between nitrogen stable isotope discrimination between predator and prey consumed (∆^15^N) and the prey stable nitrogen isotope composition (dietary-δ^15^N) estimates for each shark species based on the widely reported ∆^15^N-dietary δ^15^N relationship [7,8,40,67].

Precise methods for characterizing the ecological role of organisms are imperative to the management of aquatic ecosystems [10]. This is especially relevant for marine predators that have undergone global declines in recent decades, as many species occupy a relatively high trophic position, and are therefore assumed to play an important role in structuring marine communities [11–13]. The subclass Elasmobranchii (sharks, skates and rays) comprises ~450 species that inhabit a diverse range of habitats, from the deep ocean to exposed coral shelves. Species within this subclass range in size, from the dwarf lantern shark *Etmopterus perryi* (~17 cm) to the whale shark *Rhincodon typus* (~12 m) and feed on a broad prey base, from zooplankton to marine mammals. This considerable diversity together with the reported inverse ∆^15^N-dietary δ^15^N relationship, would suggest that variable diets among species within this subclass will result in variable mean dietary δ^15^N values (e.g., crustacean dominated diet vs. elasmobranch dominated diet) that would ultimately drive variable ∆^15^N values (Figure 1). To date, two experimentally derived ∆^15^N values are available for elasmobranchs; a ∆^15^N of 2.3‰ for large sharks fed a fish diet [14] and a ∆^15^N of 3.7‰ for a small shark fed a diet of squid [15]. These shark-specific ∆^15^N values are markedly different and when applied to dietary and/or food web analyses would result in vastly different inferences dependent on the ∆^15^N selected. This highlights the importance of considering the diet (including protein quality and quantity) of the consumer species examined to ensure the most accurate interpretation of stable isotope data for these purposes. 

 Our objective was to conduct an assessment of a range of reported ∆^15^N values for describing the predator-prey relationships of four shark species that feed at different trophic levels (i.e., different mean δ^15^N dietary values) and in so doing, determine the most applicable surrogate ∆^15^N value for describing predator-prey relationships within this speciose group. Given the logistical and ethical complexities of controlled feeding experiments on large predators, field sampling to derive consumer and prey isotope values in conjunction with novel ellipse estimates were used to assess appropriate ∆^15^N values for four species that have well characterized diets from stomach content analysis; bonnethead (*Sphyrna tiburo*), Atlantic sharpnose (*Rhizoprionodon terraenovae*), bull (*Carcharhinus leucas*) and white (*Carcharodon carcharias*) shark. The broad range of predator-prey relationships represented by the trophic assessment of these four species presents the opportunity to assess whether species-specific diets that range from a low (i.e., bonnethead preying on crustaceans) to a high (i.e., white preying on elasmobranchs and marine mammals) δ^15^N value affect the choice of which ∆^15^N value to use. 

## Materials and Methods

### Ethics Statement

All large sharks, rays and dolphins sampled in South Africa were found deceased on capture in beach protection nets and no animals were sacrificed for this study. All live net-caught sharks and rays were tagged and released according to the KwaZulu-Natal (KZN) Sharks Board tag and release protocols. Cape fur seals were sampled from animal strandings and regional culls undertaken by Marine and Coastal Management (MCM) at the Department of Environmental Affairs and Tourism, South Africa. Permission to use white shark and cape fur seal muscle tissue samples was granted by the KZN Sharks Board and MCM, respectively and samples were exported in strict accordance with the Convention on International Trade in Endangered Species of Wild Fauna and Flora (CITES) requirements (CITES South African Permit No. 106704 and 106627). All sharks, fish and invertebrates sampled in Georgia and additional sharks, dolphins, fish and invertebrates from South Africa are non-threatened species and are not listed on CITES and therefore export permits were not required. Sampling was undertaken under the authority of the Marine Fisheries Section, Coastal Resources Division, Department of Natural resources, Georgia, MCM, Cape Town, South Africa and the Oceanographic Research Institute, Durban, South Africa. All vertebrate work was conducted in accordance with the University of Windsor’s Animal Use and Care Guidelines (AAUP #07-13) and respective policies of the Department of Natural Resources, Georgia, the KZN Sharks Board, MCM, South Africa and the Oceanographic Research Institute, South Africa.

### Sample collection

 Predator (four focal shark species) and prey species (e.g., invertebrates, fishes, mammals) with the exception of cape fur seals (see below) were sampled from two study sites, the estuaries of coastal Georgia, USA and from the continental shelf of KwaZulu-Natal (KZN), South Africa. All sampling of predators and prey was conducted over the same time frames. In Georgia, invertebrates and teleosts were sampled via otter trawl during scientific research cruises, and bonnethead and Atlantic sharpnose sharks were sampled from incidental captures from shallow water bottom-set longlines (see 16). In KZN invertebrates and teleosts were sampled from three main sources: (i) by-catch in the shallow water prawn trawl fishery, (ii) organized spear fishermen catches and (iii) organized recreational fishermen/scientific catches. All elasmobranch species including bull and white sharks and delphind species were sampled from incidental captures in beach protection nets along the KZN coast [17]; cape fur seals were sampled from animal strandings and regional culls at colonies around the Eastern and Western Cape where white sharks are known to seasonally reside [18–20]. Immediately following capture, a muscle tissue sample was excised from prey species and stored frozen (-20°C) as follows: from the claws and tails of invertebrates, from the dorsal section anterior to the dorsal fin in teleosts and sharks, from the mid-wing section of batoids, and from the mid-dorsal region of seals and delphinids. For the four focal shark species, all individuals were measured (TL–total or PCL–precaudal length) and white muscle tissue was excised anterior to the first dorsal fin and stored frozen (-20°C). 

### Stomach Content and Stable Isotope Data

To characterize the diet of the four focal species, we used a combination of published literature and stomach contents sampled from animals included in this study. For bonnethead and Atlantic sharpnose sharks, stomach content data were quantified following standard methods (see 21,22, M. Fritts unpublished data). For bull and white sharks, quantified stomach content data from South African animals were taken from Cliff and Dudley [23] and Hussey et al. [24], respectively. The importance of (i) functional prey groups (e.g., crustacean, mollusk, teleost, elasmobranch, mammal) and (ii) individual prey items (identified to species level where possible) to the diet of each shark was assessed using four commonly applied dietary indices: (i) %W, the weight contribution of a prey item, (ii) %F, the frequency of occurrence of each prey item, (iii) %N, the numbers of each prey item and (iv) %IRI, the relative importance of the prey item. Specifically, %W was calculated as the weight of each prey item divided by the total weight of prey items from an individual stomach, %F was calculated as the number of stomachs containing a prey item divided by the total number of stomachs containing prey and %N was calculated as the number of each prey item divided by the total number of prey items. The index of relative importance is a compound index that incorporates the previous three indices, expressed as IRI = %F (%N + %W). This product is then expressed as a percentage (%IRI) by dividing the total IRI for each prey item by the total IRI for all prey items [22]. From the complete stomach content data (for complete diet analysis see Cliff and Dudley [23]; Hussey et al. [24]; M. Fritts unpublished data), the five most commonly occurring prey items in the diet of each shark [referenced as principal prey (PP) hereafter] were determined based on the highest percent contribution by the combination of %W, %F, %N and %IRI for each prey item (Table 1). 

**Table 1:** Summarized functional prey groups and the principal prey (PP) of bonnethead *Sphyrna tiburo*, Atlantic sharpnose *Rhizoprionodon terraenovae*, bull *Carcharhinus leucas*, and white *Carcharodon carcharias* shark identified from stomach content data (see 9,23 M. Fritts unpublished data).

	*Sphyrna tiburo*				*Rhizoprionodon terraenovae*				*Carcharhinus leucas*				*Carcharodon carcharias*			
	TL = 39–47 cm				TL = 34–49 cm				PCL = 145–208 cm				PCL = 185–234.9 cm			
	%F	%N	%W	%IRI	%F	%N	%W	%IRI	%F	%N	%W	%IRI	%F	%N	%W	%IRI
**MAMMAL**									**7.7**	**2.6**	**7.5**	**0.6**	**34.1**	**4.8**	**24.5**	**27.4**
Common dolphin (*Delphinus delphis*)													0.8	0.1	5.5	0.4^b^
Cetacea									4.3	1.4	5.1	3.3^d^				
Seal (Pinnipedia)													7.0	1.0	5.4	4.1^c^
**ELASMOBRANCH**									**57.5**	**20.7**	**65.5**	**40.6**	**42.6**	**7.2**	**46.3**	**62.3**
Guitarfish (Rhinobatidae)									9.5	3.2	17.8	11.4^c^				
Dusky/Sharpnose shark (Carcharhinidae)									6.2	2.3	8	3.3^a^				
Bull/Blue/Honeycomb stingray (Dasyatidae)									1.5	0.6	2.2	0.2^b^				
Dusky shark(*Carcharhinus obscurus*)													10.9	2.6	18.1	20.9^a^
Milk shark(*Rhizoprionodon acutus007A*)													2.3	0.3	2.52	0.61^d^
Spotted eagle ray (*Aetobatus narinari*)													0.8	0.1	3.2	0.2^e^
**TELEOST**	**7.5**	**3.4**	**4.5**	**0.6**	**47.8**	**33.2**	**58.5**	**49.3**	**71.1**	**74.5**	**25.4**	**58.5**	**22.4**	**5.1**	**5.4**	**6.4**
Spot (*Leiostomus xanthurus*)					2.3	1.3	15.3	1.1^a^								
Star drum (*Stellifer lanceolatus*)					2.3	1.3	13.7	1^b^								
Spotted grunter (*Pomadasys commersonnii* )									4.8	1.7	1.6	1.9^e^				
**CRUSTACEAN**	**122.5**	**58.6**	**70.8**	**46**	**38.7**	**23.9**	**28.2**	**17.8**	**5.3**	**1.8**	**0.1**	**0.1**				
Blue crab (*Callinectes* sp.)	22.5	11.5	18.2	12.2^a^												
Lady crab (*Ovalipes ocellatus*)	7.5	4.6	13.7	2.5^b^												
Hermit crab (*Pagurus* sp.)	10	4.6	5	1.7^d^	6.8	5.3	7	2.4^d^								
Shrimp (Penaeidae)	15	6.9	10.4	4.7^c^	18.2	10.7	15.3	13.2^c^								
Mantis shrimp (Stomatopoda)	10	4.6	6.3	2^e^												
**MOLLUSK**					**2.3**	**1.3**	**11.3**	**0.8**	**5.8**	**2.0**	**3.8**	**0.1**	**23.3**	**5.9**	**0.1**	**3.9**
Squid sp. (Teuthoidea)					2.3	1.3	11.3	0.8^e^								

Data are percent frequency of occurrence (%F), number (%N), weight (%W) and the index of relative importance (%IRI). TL represents total length; PCL represents precaudal length. Subscripts represent PP species in Figure 4.

Note: Principal prey (PP) do not contribute 100% to the total of each functional prey group. A component of the diet of each of each shark species consists of unidentified species (see 9,23).

Stable carbon and nitrogen isotopes were analyzed in muscle tissue of the four predators and the determined PP species. Additional prey species identified in the diet of each species, where samples were available from respective study regions and time periods, were also analyzed. To ensure no bias of the maternal isotope signature, only bonnethead (*n* = 11; 39–47 cm TL) and Atlantic sharpnose (*n* = 6; 34–49 cm TL) sharks that were characterized as juveniles, yet older than umbilical scar stage 5 were used [19]. To avoid complications of ontogenetic diet shifts, defined size classes of bull (*n* = 11) and white (*n* = 16) sharks were selected; sub-adults/adults ranging in length from 145–208 cm PCL and sub-adults ranging in length from 185–235 cm PCL, respectively. For complete stable isotope sample preparation, lab analyses and analytical precision, see Olin et al. [16] and Hussey et al. [25]. Briefly, muscle tissues were sub-sampled from all shark and prey species (~1.0 g), freeze-dried for 48 h, ground and lipid extracted by twice agitating the ground tissue in 2:1 chloroform/methanol solution for 24 h and decanting the solvent (modified method outlined in [26]). The relative abundances of carbon (^13^C/^12^C) and nitrogen (^15^N/^14^N) were determined on ~0.5–1.0 mg sub-samples on a Thermo Finnigan Delta^Plus^ mass spectrometer (Thermo Finnigan, San Jose, CA, USA) coupled with an elemental analyzer (Costech, Valencia, CA, USA). Analytical accuracy was 0.14‰ for δ^15^N data and 0.05‰ for δ^13^C data based on a single run of NIST standard sucrose (NIST 8542; *n* = 13) and ammonium sulfate (NIST 8547; *n* = 13). 

**Figure 2:**
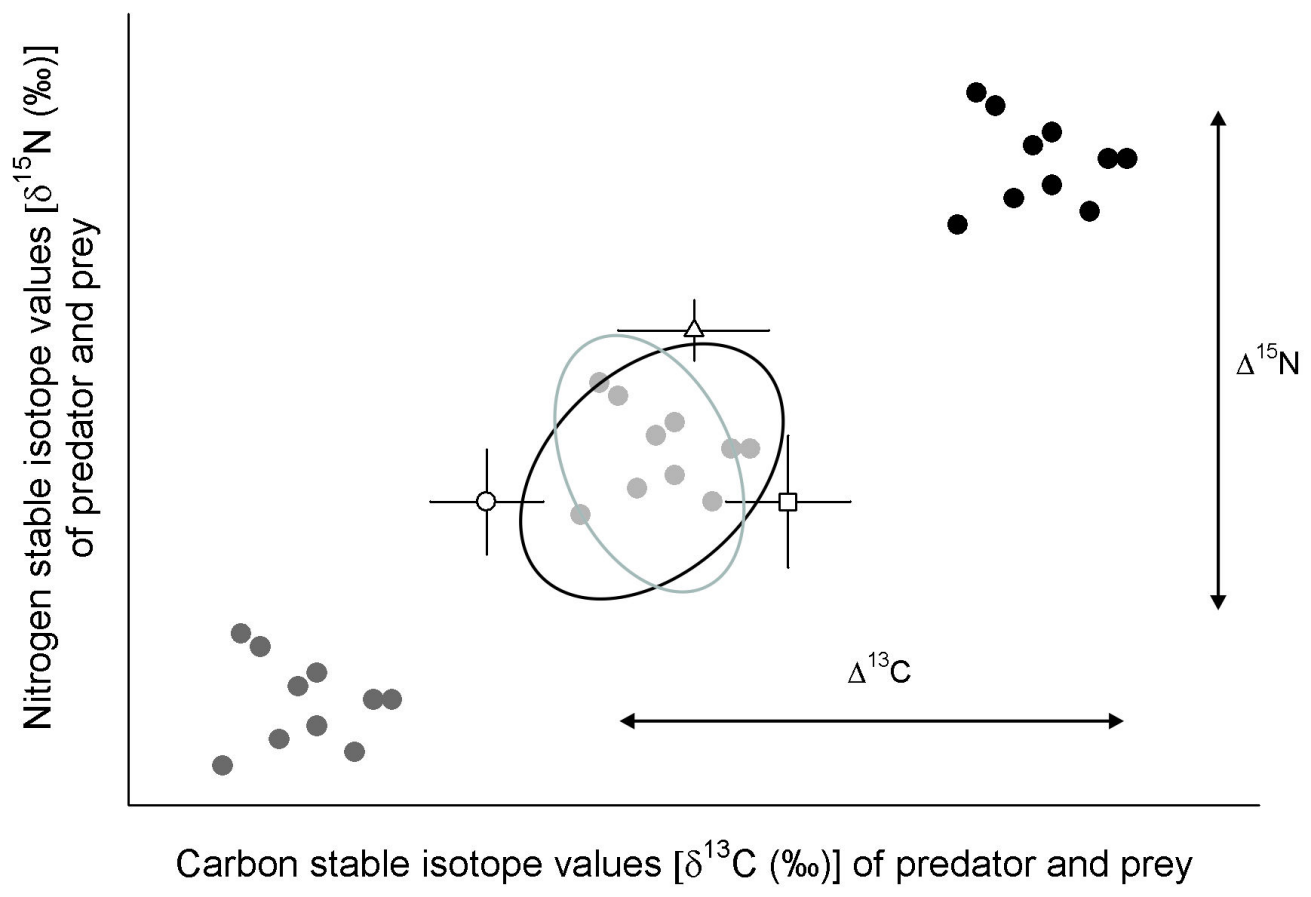
Illustration of the expected relationship between stable isotope values of a predator and its’ prey in mixing space [27,28], employing the Bayesian approach of Jackson et al. [29], centered on multivariate ellipse based metrics. In choosing discrimination factor (∆^15^N and ∆^13^C) values, it would be expected that the δ^15^N and δ^13^C values of the predator after adjustment to specific ∆^15^N and ∆^13^C values should overlay or fall within the range of δ^15^N values of the PP it consumes (see Table 2), indicating a best-fit scenario between predator and prey [ellipses represent prey (black) and predator (gray) respectively]. Black points represent δ^13^C and δ^15^N values of a predator, gray points (light and dark) represent adjusted-δ^13^C and adjusted-δ^15^N values with two different ∆^15^N and ∆^13^C values. White shapes represent mean (± variance) of prey species.

To examine whether ∆^15^N values varied among species and were dependent on the diet-δ^15^N value for a particular shark species, a range of ∆^15^N values (and associated ∆^13^C) were selected from the published literature (Table 2). The δ^15^N and δ^13^C data for each shark species were then adjusted using each of the four chosen ∆^15^N and ∆^13^C pairs from Table 2, by subtracting the specific ∆^15^N and ∆^13^C value from each individual predator’s δ^15^N and δ^13^C value, respectively. For each shark species, four δ^15^N vs. δ^13^C bi-plots were then constructed for each ∆^15^N- and ∆^13^C-adjusted shark isotope data and overlaid over the raw prey stable isotope data. Raw shark isotope data were also plotted over raw prey isotope data. This enabled a visual assessment of the ∆^15^N and ∆^13^C-adjusted shark isotope values, i.e., it would be expected that the best fit ∆^15^N and ∆^13^C-adjusted shark isotope values would fall within the range of the PP it consumes (Figure 2), following Philips and Gregg [27] and Fry [28].

**Table 2:** Diet-tissue discrimination factor (∆^13^C and ∆^15^N are the changes in isotope ratios (δ^13^C and δ^15^N) between predator and prey, calculated as δ^b^X_predator_ - δ^b^X_prey_ = ∆^b^X_predator-prey_) derived from controlled feeding experiments specific to elasmobranchs^1^ and those commonly accepted from the literature^2^.

Diet-Tissue Discrimination Factor ± SD (‰)		
∆^13^C	∆^15^N	Source
0.9 ± 0.3	2.3 ± 0.2	[14]^1^
0.8 ± 0.1	2.8 ± 0.1	[7]^2^
0.4 ± 1.3	3.4 ± 1.0	[3]^2^
1.7 ± 0.5	3.7 ± 0.4	[15]^1^

### Data Analysis

To determine whether the δ^15^N value of the diet was different among the four predators, an analysis of variance (ANOVA) followed by a Tukey’s HSD post-hoc analysis was performed on the δ^15^N data for the five-combined PP of each shark. To then quantify the best-fit ∆^15^N for each shark species, standard ellipses around the isotopic values of PP and ∆^15^N and ∆^13^C-adjusted predator combination were created using a Bayesian approach centered on multivariate ellipse-based metrics (SIBER–Stable Isotope Bayesian Ellipses; [29]). To reduce the influence of isotopic variability associated with variable sample sizes, we grouped PP into functional prey groups according to Table 1. This grouping then allowed for qualitative comparisons among predator and prey isotope and stomach content contributions. The adopted SIBER metric analyses were adapted from community–level metrics developed originally by Layman et al. [30] based on the mean δ^13^C and δ^15^N of all species in a community. Briefly, the standard ellipse represents a set of bivariate data calculated from the variance and covariance of the *x* and *y* data. The ellipse contains approximately 40% of the data and therefore represents the core niche or dietary isotopic space [31]. This approach generates standard ellipse areas (SEA), which are designed to identify differences in isotopic space between characteristic members of a population and account for populations with different sample sizes [29]. Following Jackson et al. [29], SEA was graphically expressed using a corrected SEA_C_ measurement. The area of overlap between predator and functional prey group ellipses was then calculated using the Stable Isotope in R package (SIAR; [32]) in R 2.13.0 [33]. Our expectation was that the greatest overlap between predator and PP ellipses best characterizes the predator-prey relationship and as a result, the most suitable ∆^15^N for each species. In addition, the overlap of predator ellipse with functional prey group ellipses would rank in terms of importance to diet from stomach content data, providing further confidence in the selection of the most suitable ∆^15^N value. The above analyses were repeated, using isotopic values from a broader range of prey species identified in the diet of each shark, i.e., those species identified from stomach contents with available isotopic values (number of additional prey species: bonnethead = 11; Atlantic sharpnose = 15; bull = 41; and white = 25). This enabled a comparison of ellipse overlap between predator vs. functional prey groups (based on PP) and predator vs. broader diet for each species to illustrate the importance of considering the number of dietary items and their relative importance to diet for confidence in this approach.

## Results

**Figure 3:**
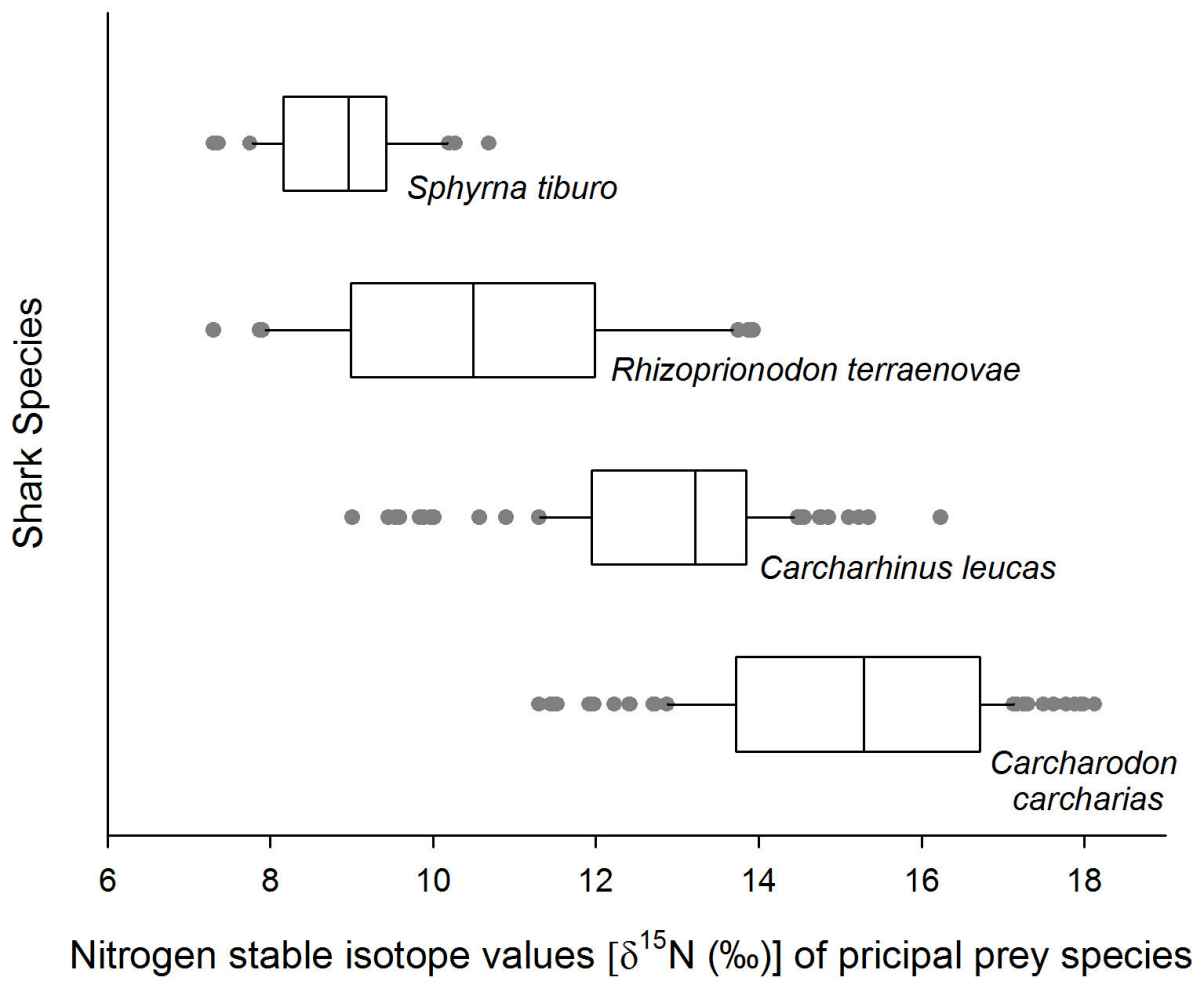
Box plots representing the δ^15^N values of all of the PP derived from stomach content data of the bonnethead *Sphyrna tiburo*, Atlantic sharpnose *Rhizoprionodon terraenovae*, bull *Carcharhinus leucas*, and white *Carcharodon carcharias* shark.

In terms of %W, %F, %N and %IRI crustaceans were the principal diet component of the bonnethead, teleosts for the Atlantic sharpnose, elasmobranchs and teleosts for the bull shark, and elasmobranch and marine mammals for the white shark (Table 1). The mean diet δ^15^N value for each shark species based on PP ranged from low in the bonnethead shark (8.8 ± 0.9‰; Figure 3), to high in the white shark (15.1 ± 1.7‰; Figure 3), and differed significantly among the four species (*F*_*3,322*_ = 177.4, *P* < 0.0001; Figure 3). 

**Figure 4:**
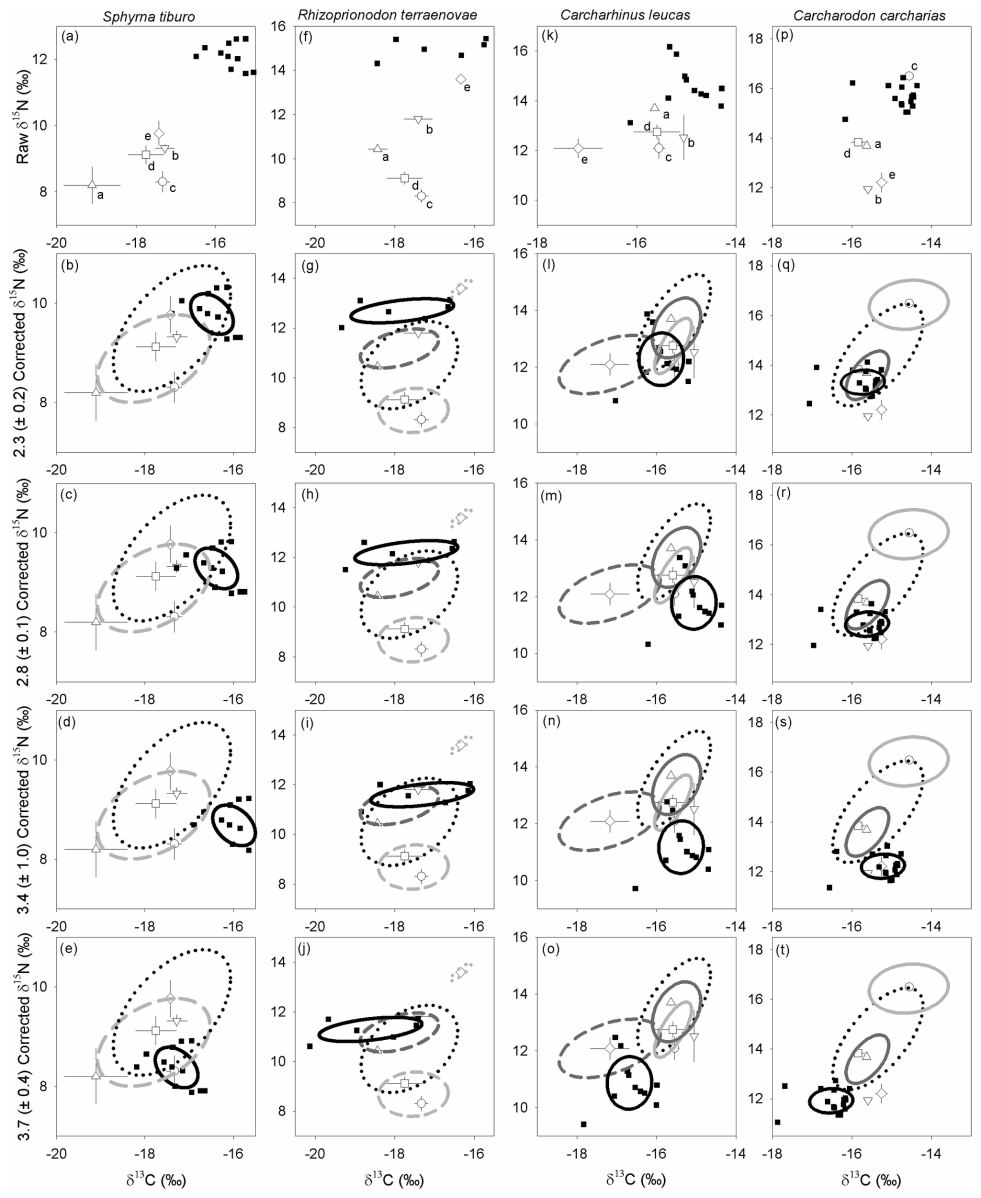
Dual-plot of individual predator (■) and mean (± SD) δ^13^C and δ^15^N values of the PP for each predator ((a), (f), (k), (p); see Table 1). Standard ellipse areas corrected for sample size (SEA_c_) of sharks (solid black) and PP functional prey groups (Crustacean, dashed light gray; Mollusk, dotted light gray; Teleost, dashed dark gray; Elasmobranch, solid dark gray; Mammal solid light gray), and the broader diet (dotted black) following Jackson et al. [29]. Note different scales on the x- and y-axes in each species.

The bonnethead shark exhibited the most enriched raw δ^15^N values relative to PP, while the white shark exhibited the least enriched raw δ^15^N values relative to PP (Figure 4a, f, k, p). For the bonnethead shark that had the lowest δ^15^N diet value, the largest ∆^15^N of 3.7‰ produced both the greatest visual and ellipse overlap between predator and the functional prey group identified from stomach contents (Figure 4e; Table 3). The most suitable ∆^15^N for the Atlantic sharpnose based on ellipse overlap was lower than the bonnethead, a ∆^15^N value of 3.4‰ (Figure 4i; Table 3), while 2.3‰ provided the highest measure of overlap for the bull shark (Figure 4l; Table 3). For the white shark, a ∆^15^N of 2.3‰ produced the greatest area of overlap between predator and functional prey group ellipses but observation of the data suggested that the ∆^15^N for this highly carnivorous species is likely lower (Figure 4p, q; Table 3). The greatest area of overlap between ∆^15^N-adjusted predator values and functional prey group isotope values for all four shark species generally correspond with what we expected based on the contributions estimated from stomach content analyses. For example, teleosts followed by crustaceans and mollusks contributed the highest combined percentage to the diet of the Atlantic sharpnose (%W = 58.5, 28.2 and 11.3, respectively; Table 1), which corresponded to the calculated ellipse area overlap between the Atlantic sharpnose and the functional prey groups using the ∆^15^N value of 3.4‰ (Table 3). These results were further supported when including stable isotope values for additional prey items identified from the broader diet with the exception of the bonnethead shark (Figure 4b, e; Table 3).

**Table 3:** Calculated overlap of SIBER ellipses between (i) isotopic values of the PP functional prey groups (see Table 1) and (ii) the isotopic values of the broader diet of each shark derived from stomach content analyses, and the ∆^15^N- and ∆^13^C-adjusted isotopic values (see Table 2) for each shark (see Figure 4) following Jackson et al. [29].

∆^15^N Values	Predator (isotopic area)	Functional Prey Group (isotopic area)							Overlap Predator-Functional Prey Group (area)					
		Crust	Moll	Teleost	Elasmo	Mamm	All		Crust	Moll	Teleost	Elasmo	Mamm	All
*Sphyrna tiburo*														
2.3 ± 0.2	0.30	1.66					2.29		0.00					**0.16**
2.8 ± 0.1	0.30	1.66					2.29		0.00					0.03
3.4 ± 1.0	0.30	1.66					2.29		0.00					0.00
3.7 ± 0.4	0.30	1.66					2.29		**0.27**					0.01
*Rhizoprionodon terraenovae*														
2.3 ± 0.2	0.90	1.26	0.07	1.12			3.18		9.34^-16^	0.00	8.28^-16^			2.24^-16^
2.8 ± 0.1	0.90	1.26	0.07	1.12			3.18		1.05^-15^	0.00	6.00^-16^			5.01^-15^
3.4 ± 1.0	0.90	1.26	0.07	1.12			3.18		**1.51^-15^**	**5.42^-20^**	**0.41**			**0.41**
3.7 ± 0.4	0.90	1.26	0.07	1.12			3.18		1.46^-17^	0.00	0.31			0.09
*Carcharhinus leucas*														
2.3 ± 0.2	0.84			1.88	0.96	0.45	1.89				**0.01**	**0.07**	**0.24**	**0.25**
2.8 ± 0.1	0.84			1.88	0.96	0.45	1.89				0.00	2.06^-18^	1.14^-18^	2.39^-18^
3.4 ± 1.0	0.84			1.88	0.96	0.45	1.89				0.00	7.40^-18^	1.12^-17^	1.40-^17^
3.7 ± 0.4	0.84			1.88	0.96	0.45	1.89				6.70^-18^	0.00	0.00	0.00
*Carcharodon carcharias*														
2.3 ± 0.2	0.43				0.69	1.57	2.86					**0.34**	0.00	**0.40**
2.8 ± 0.1	0.43				0.69	1.57	2.86					0.06	0.00	0.05
3.4 ± 1.0	0.43				0.69	1.57	2.86					1.61^-16^	0.00	5.20^-18^
3.7 ± 0.4	0.43				0.69	1.57	2.86					0.00	0.00	1.09^-18^

The extent of overlap ranges from 0 to 1, with values closer to 1 representing more overlap. Bold values highlight the greatest overlap between predator and designated prey group.

Note: Functional prey groups are noted as follows: Crust = Crustacean; Moll = Mollusk; Elasmo = Elasmobranch; Mamm = Mammals. The broader diet (All) consists of prey species identified from stomach contents with available isotopic values from the same sampling location (# of species included: bonnethead = 11; Atlantic sharpnose = 15; bull = 41; and white = 25).

## Discussion

 Selection of appropriate ∆^15^N values for interpreting stable isotope data remains a controversial point (see 7,14,34,35). The implication of using inappropriate ∆^15^N values when calculating trophic position or undertaking mixing model analyses will result in inaccurate inferences regarding diet reconstruction and more critically, the role of an organism within aquatic food webs [36]. Species-specific ∆^15^N values have been recognized [37,38] and are particularly important given the advanced isotope modeling approaches currently being adopted to examine complex feeding behavior at both the population and individual level [30,39]. These data find that the greatest area of ellipse overlap between predator and prey was a result of selecting different ∆^15^N values that were dependent on the diet δ^15^N value, indicating that ∆^15^N values for species within the subclass Elasmobranchii vary greatly. Although considerable progress in stable isotope controlled feeding studies has been made [5,6,8], the ∆^15^N-diet δ^15^N relationship has largely been ignored based on the fact that the mechanism driving the relationship remains unknown. Despite this, our results further corroborate this relationship and future experimental analyses should focus on identifying the mechanism driving this relationship. Whether the relationship is driven by protein quantity and quality [36,40] or the variable kinematics of ^14^N and ^15^N routing and incorporation in animal tissues [37] remains unclear, but questions the use of a single ∆^15^N value for this subclass. 

 Variation in ∆^15^N values dependent on the consumers’ dietary-δ^15^N value within this subclass of species would indicate this result is ubiquitous across a broad range of taxa in both aquatic and terrestrial ecosystems. These results were consistent across shark species when comparing both isotope data of functional prey groups and the broader identified diet, the latter with the exception of the bonnethead shark. This inconsistency in ∆^15^N values of the bonnethead resulted from the inclusion of teleost prey species with higher δ^15^N values (i.e., anchovies) in the broader diet, prey species that are minor contributors to overall diet. The bonnethead shark is known to primarily feed on crustaceans with minimal contribution from teleost prey (see 41 and references therein). Our overall results were also consistent with other studies that have reported greater isotopic discrimination between predator and prey when a low δ^15^N diet is assimilated [7,40]. Indeed, Kim et al. [42], observed a higher ∆^15^N value in a controlled laboratory study on leopard sharks (*Triakis semifasciata*) following a diet switch from squid (~3.7‰; δ^15^N of diet = ~13.3‰) to tilapia (~5.5‰; δ^15^N of diet = ~7.9 ‰). Similarly, Blanchet-Aurigny et al. [43] observed higher ∆^15^N values for brittle stars *Ophiocomina nigra* and *Ophiothrix fragilis* fed a diet of mussels and/or macroalgae (2-4‰) compared to a diet of fish (~-1‰). This demonstrates that ∆^15^N values can vary within a species dependent on the dietary-δ^15^N value the animal consumes, in agreement with previous work [6,44]. If ∆^15^N values vary within a species, dependent on the dietary-δ^15^N value, it would be expected that ∆^15^N values will vary among species within a subclass that have diverse diets as our data indicate. 

Compound specific stable isotope analysis of amino acids (AA-CSIA) has proven to be a complimentary method for estimating ∆^15^N values within organisms (trophic enrichment factor —TEF). Initial experimental work suggested a TEF value of 7.6‰ was universal across species and taxa [45], similar to the widely applied value of 3.4‰ used in bulk stable isotope analysis [3]. Recently published data on marine mammals fed high δ^15^N diets exhibited TEFs that were lower than previously observed (4.3 vs. 7.6‰) [46]. These AA-CSIA data are consistent with the relationship observed in bulk stable isotope analysis [7,8,14] and suggest that TEF estimates may vary dependent on the diet δ^15^N value. Knowledge of diet ascertained through stomach content data remains critical for guiding isotopic assumptions on ∆^15^N values and thereby improving the efficiency of yielded results from modeling exercises, for example mixing models [47,48]. 

 Estimating trophic position and the application of mixing models for dietary reconstruction are particularly vulnerable to the basic assumption of which ∆^15^N value to use [6,49], with consequences not only for ecological investigations but for management decisions [49]. This point is pertinent given the promoted use of stable isotopes to investigate threatened and endangered species [49]. Specific to the white shark, stable isotopes have recently been used to derive data on feeding behavior, movement and their role in food webs, overcoming the logistical difficulties and conservation concerns of studying these large predators. For example, Carlisle et al. [39] developed a novel isotope mixing model integrating both movement data and tissue turnover rates to estimate the relative importance of different focal habitat areas and associated prey to white shark diet, and to elucidate migratory behavior. Whereas Kim et al. [50] generated ontogenetic isotopic profiles of individual white sharks using serial sampling of vertebrae. In addition, a number of studies have presented trophic position estimates for a range of elasmobranch species based on stable isotopes (e.g., [51–53]). The integrity of data interpretation in all the above studies and the broader isotopic literature, which may ultimately influence management decisions such as conservation status listing (i.e., CITES) and the designation of critical habitat and/or marine protected areas for predator and/or prey, is dependent on assumptions over ∆^15^N values. This is of particular importance given the recognized use of food web models by resource managers that may incorporate parameter estimates from stable isotope analyses (e.g., [54]). Future modeling exercises focused on the trophic ecology of these large marine predators using isotopic data should consider evaluating the sensitivity of predictions generated from models to different fractionation assumptions (e.g., [55]).

It is possible that several factors may have biased our observed ∆^15^N-diet δ^15^N results. These include (i) the use of stomach content data for ranking the importance of prey species, (ii) movement of predators between isotopically distinct food webs, (iii) ontogenetic diet shifts, and, (iv) temporal variability in prey and baseline isotopic values. Stomach content data is often criticized for only providing a ‘snapshot’ of a species diet [56], biases largely resulting from varying rates of prey digestion, empty stomachs and limited sampling in spatial and temporal scope. However stomach content analysis remains one of the most important measures of an animal’s feeding ecology providing data on the actual species consumed [57] and when coupled with stable isotopes analysis have resulted in significant ecological insights, for example, reef community connectivity [55], sized-based interactions [52] and niche overlap [58] that would have been difficult to infer using a single approach. The high degree of isotopic ellipse overlap between predators and prey, ranked by importance from stomach content data, would indicate our dual approach was valid. Sharks are known to be mobile predators that can feed over variable spatial and temporal domains [39]. For this reason we targeted specific size classes of our focal species to avoid confounding ontogenetic effects. For example, juvenile bonnethead and Atlantic sharpnose sharks have relatively small home-ranges, largely inhabiting coastal bays and estuaries [59,60] and sub-adult/adult bull sharks show high site fidelity [61], with only occasional large scale movements in coastal habitats [62,63]. In the case of sub-adult white sharks used in this study, individuals make directed seasonal movements between two distinct regions, the Western Cape and KZN. Considering this point, prey species were sampled in KZN and cape fur seals, the principal diet item from the Cape region were sampled, accounting for potential spatial bias in prey isotope values. Moreover, isotopic variability across space and time might be expected to enhance variation within functional prey groups (i.e., larger ellipse areas) and therefore lead to greater overlap between predator and prey. This was not observed in our analyses. Finally, integration of prey isotopes values in shark muscle tissue is on the order of <12 months (e.g., [34,64]). Consequently shark muscle tissue isotope values represent foraging over multiple seasons and locations, thereby minimizing the influence of these variables on the results of our analysis. 

In conclusion, variable ∆^15^N values provided a more accurate representation of the predator-prey isotopic relationship for sharks rather than the application of a single ∆^15^N for species that feed across different trophic levels or dietary δ^15^N. Controlled feeding experiments to derive experimental ∆^15^N values for large predators are challenging due to the ethical issues of feeding animals a single diet item and therefore require in the vast majority of cases, researchers to compromise and apply a ∆^15^N value based on studies of either ecologically or taxonomically similar species [49]. Moreover, most organisms such as sharks feed on multiple prey items. Consequently experimental derived values based on a single diet item could be inaccurate or misleading. Given the strength of empirical work to date, we suggest that evaluation of a range of ∆^15^N values is a necessary consideration for future trophic and diet reconstruction analyses and is essential for improving the understanding of a species’ ecological role in its’ environment and to enhance associated conservation and management actions. With the recent advancements in mathematical modeling in Bayesian frameworks [65,66], whereby the use of stable isotopes to examine predator-prey relationships has become more precise and approaches more refined, the novel application of SIBER that incorporates predator and prey isotopic variability combined with stomach content data provided a greater level of confidence, in the context of this analysis, in choosing an appropriate ∆^15^N value. 
